# The biogenesis and biological function of PIWI-interacting RNA in cancer

**DOI:** 10.1186/s13045-021-01104-3

**Published:** 2021-06-12

**Authors:** Silu Chen, Shuai Ben, Junyi Xin, Shuwei Li, Rui Zheng, Hao Wang, Lulu Fan, Mulong Du, Zhengdong Zhang, Meilin Wang

**Affiliations:** 1 Jiangsu Cancer Hospital, Jiangsu Institute of Cancer Research, The Affiliated Cancer Hospital of Nanjing Medical University, 101 Longmian Avenue, Nanjing, 211166 Jiangsu People’s Republic of China; 2 Department of Environmental Genomics, Jiangsu Key Laboratory of Cancer Biomarkers, Prevention and Treatment, Collaborative Innovation Center for Cancer Personalized Medicine, Nanjing Medical University, Nanjing, China; 3 Department of Genetic Toxicology, The Key Laboratory of Modern Toxicology of Ministry of Education, Center for Global Health, School of Public Health, Nanjing Medical University, Nanjing, China; 4 Department of Biostatistics, Center for Global Health, School of Public Health, Nanjing Medical University, Nanjing, China; 5 Suzhou Municipal Hospital, Gusu School, The Affiliated Suzhou Hospital of Nanjing Medical University, Nanjing Medical University, Nanjing, China

**Keywords:** piRNAs, Biogenesis, Database, Cancer hallmarks, Biomarker

## Abstract

Small non-coding RNAs (ncRNAs) are vital regulators of biological activities, and aberrant levels of small ncRNAs are commonly found in precancerous lesions and cancer. PIWI-interacting RNAs (piRNAs) are a novel type of small ncRNA initially discovered in germ cells that have a specific length (24–31 nucleotides), bind to PIWI proteins, and show 2′-O-methyl modification at the 3′-end. Numerous studies have revealed that piRNAs can play important roles in tumorigenesis via multiple biological regulatory mechanisms, including silencing transcriptional and posttranscriptional gene processes and accelerating multiprotein interactions. piRNAs are emerging players in the malignant transformation of normal cells and participate in the regulation of cancer hallmarks. Most of the specific cancer hallmarks regulated by piRNAs are involved in sustaining proliferative signaling, resistance to cell death or apoptosis, and activation of invasion and metastasis. Additionally, piRNAs have been used as biomarkers for cancer diagnosis and prognosis and have great potential for clinical utility. However, research on the underlying mechanisms of piRNAs in cancer is limited. Here, we systematically reviewed recent advances in the biogenesis and biological functions of piRNAs and relevant bioinformatics databases with the aim of providing insights into cancer diagnosis and clinical applications. We also focused on some cancer hallmarks rarely reported to be related to piRNAs, which can promote in-depth research of piRNAs in molecular biology and facilitate their clinical translation into cancer treatment.

## Introduction

Small non-coding RNAs, a type of non-coding RNA (ncRNA), are divided into several main types, such as microRNAs (miRNAs), small nuclear RNAs (snRNAs), small nucleolar RNAs (snoRNAs), PIWI-interacting RNAs (piRNAs), and transfer RNAs (tRNAs) [1]. Numerous small ncRNAs have been widely reported to have vital roles in the pathogenesis and treatment of human cancers. Among these, piRNAs, which have a length of 24–31 nucleotides, are the least investigated class of small RNAs. The precursors of piRNAs are derived from tandem repeat sequences called piRNA clusters and form a mature piRNA/PIWI complex via two routes dependent or independent “Ping-Pong” amplification pathways [2]. piRNAs were initially identified as highly expressed in germline cells and able to silence transposons to maintain genome integrity and thereby protect the genome against transposon-induced defects in gametogenesis and fertility [3, 4]. In addition, it has been reported that piRNAs are also widely expressed in somatic cells [5] and play regulatory roles, such as silencing the transcriptional gene process, regulating translation and mRNA stability, maintaining stem cell functions, and interacting with multiple proteins [6]. These affluent regulatory mechanisms of piRNAs in both germ and somatic cells greatly expand the scope of the existence of piRNAs and attract the interest of scientists. An increasing number of researchers are attempting to explore the mechanism of piRNAs in tumorigenesis and their application prospects in tumor diagnosis and treatment.

It has been reported that piRNAs are involved in the occurrence of numerous cancers, including renal cell carcinoma, prostate carcinoma, and diffuse large B-cell lymphoma [7–10]. In addition, piRNAs act as potential diagnostic markers in the early diagnosis for lung cancer, colorectal cancer, and gastric cancer [11–16]. piRNAs are also important prognostic indicators for kidney cancer and colorectal cancer after treatment [7, 17–19]. piRNAs have also been found to affect the survival of tumor cells exposed to chemotherapy drugs and may act as novel therapeutic targets for human cancer [20–22]. Therefore, exploring the molecular mechanism of piRNAs in the process of tumorigenesis can provide new insights into therapeutic targets and research on drug resistance.

This review will systematically summarize the research progress toward understanding the biogenesis and biological functions of piRNA and the relevant bioinformatics databases with the aim of providing information for cancer diagnosis and therapeutics.

## Biogenesis mechanisms and characteristics of piRNAs

### The generation of primary piRNAs

**Fig. 1:**
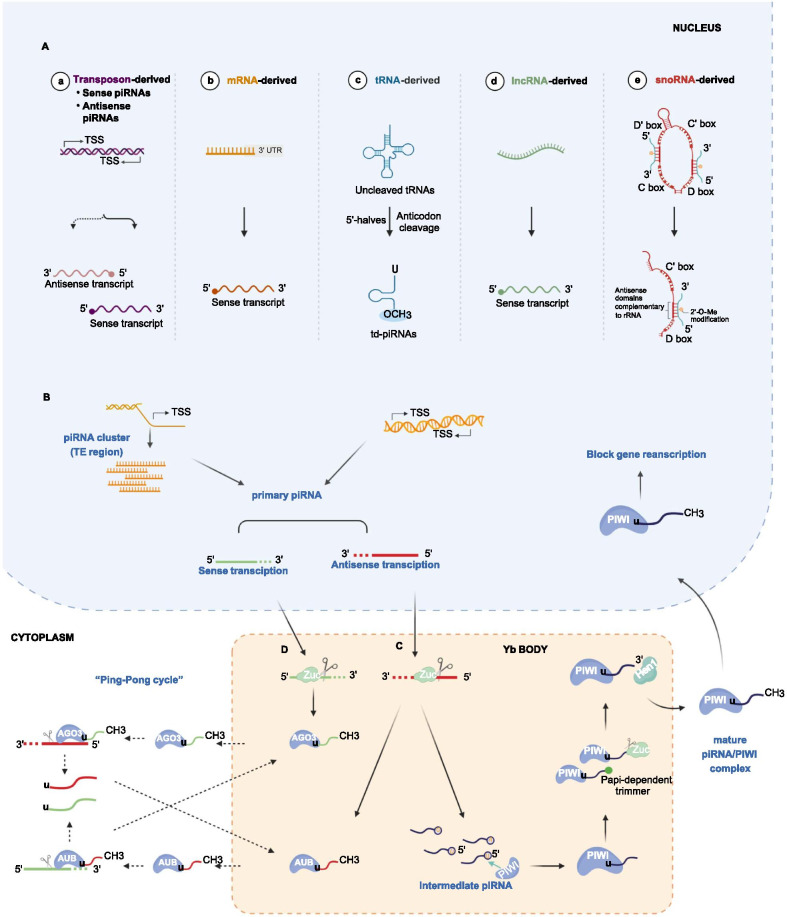
Biogenesis mechanisms and characteristics of piRNAs. (**A**) Five origins of primary piRNAs. It has been confirmed that the generation of piRNAs can be divided into five groups depending on their origin: (a) transposon-derived, (b) mRNA-derived, (c) tRNA-derived, (d) lncRNA-derived, and (e) snoRNA-derived piRNAs. (**B**) Sense and antisense transcripts of the primary piRNAs are produced by small nucleotide sequences from clusters of piRNA genes. (**C**) Primary piRNAs are cleaved by Zuc in the YB body, and the 5′ fragments of the intermediate sequence interact with a PIWI protein to form the piRNA/PIWI complex and thus intermediate piRNAs. The 2′-hydroxyl at the 3′-end of the intermediate piRNA is methylated by Hen1 to form a mature piRNA/PIWI complex. (**D**) The primary piRNA binds to AGO3 or AUB proteins through the “ping-pong cycle” to form complexes in the nucleus. Given the complementary piRNA sequences of these two complexes, the piRNA/AGO3 complex splits target RNA in the cytoplasm to produce corresponding new sequences, which can then be used as substrates to bind to AUB proteins in the nucleus to form new piRNA/AUB complexes. *TSS* transcription start site; *Zuc* zucchini

Research on piRNA biogenesis mechanisms is increasing but remains insufficient [23–25]. Primary piRNAs originate from specific genomic loci called piRNA clusters in which piRNAs line up end-to-end or overlap slightly in a strand-specific manner, and a large fraction of piRNAs can be uniquely mapped to these clusters [2]. More than 90% of the piRNAs are directly derived from the piRNA clusters [3, 26]. In general, it has been confirmed that piRNAs can be classified into five groups based on origin: transposon-derived, mRNA-derived, transfer RNA (tRNA)-derived, long noncoding RNA (lncRNA)-derived, and snoRNA-derived piRNAs (Fig. 1A). Transposon-derived piRNAs originate from single-strand clusters transcribed, generating both sense and antisense piRNAs [27]. piRNAs derived from mRNAs originate from mRNA 3′ untranslated regions (3′UTRs) and recognize the mRNA from which they are processed [28]. tRNA-derived piRNAs are produced from 5′-tRNA halves and not mature tRNAs [29–31]. piRNAs derived from lncRNAs are produced from lncRNA exonic regions [32]. snoRNA-derived piRNAs are processed from snoRNAs cleaved by the middle of their sequences, containing C/D boxes and C'/D’ boxes [33, 34]. The C box (UGAUGA) and D box (CUGA) are two conserved motifs unique to snoRNA and indispensable parts of snoRNA positioning and processing, while the C’ box and D’ box are called antisense boxes and located in the middle of the C/D box. These boxed C(C′)/D(D′) motifs are critical for protein binding and required for the function of this class of piRNAs [35]. However, the generation and shearing mechanisms and auxiliary proteins of primary piRNAs are still unclear and need further study.

### Two routes produce mature piRNAs

Precursor piRNAs are produced from genomic sources and then transferred from the nucleus to the cytoplasm. After being cleaved and modified, the intermediate piRNAs then form mature piRNAs in a complex with PIWI proteins [36]. The processes are complex and have not been intensively studied, but the following two major pathways have been described: the primary amplification pathway and the “Ping-Pong” amplification pathway (Fig. 1).

The primary amplification pathway produces sense and antisense transcripts from small nucleotide sequences from clusters of piRNA genes (Fig. 1B). After being exported to the Yb body in the cytoplasm, these primary piRNAs are cleaved by Zucchini (Zuc), an endoribonuclease enzyme [37, 38]. Then, the 5′ fragment of the intermediate sequences interacts with a PIWI protein to form a piRNA/PIWI complex, known as the intermediate piRNAs. An exonuclease or Zuc trims the 3′-end of the piRNA to its mature length, and the 2′-hydroxy group at the 3′-end is methylated by HEN1 to subsequently yield the mature piRNA/PIWI complex [24, 27, 39]. Finally, the complexes block target gene transcription by migrating back to the nucleus (Fig. 1C) [40, 41].

The “Ping-Pong” amplification pathway serves as the second mechanism for producing many piRNAs in the cytoplasm (Fig. 1D) [27, 42]. piRNAs bind to AGO3 or AUB proteins rather than PIWI proteins to form piRNA/AGO3 or piRNA/AUB complexes in the nucleus. The AUB-bound piRNAs showed a 5′ bias for U, whereas AGO3-bound piRNAs featured an A at position 10 [43, 44]. These two types of complexes contain mutually complementary piRNA sequences. Thus, a piRNA/AGO3 complex will cut the target RNA to synthesize a new sequence in the cytoplasm and then serve as a substrate for forming a piRNA/AUB complex with the AUB protein in the nucleus. Similar to the previous process, piRNA/AUB protein complexes will migrate from the nucleus to the cytoplasm and cut the complementary RNA sequence, resulting in the generation of new RNA substrates that form new piRNA/AGO3 complexes [45]. This interesting amplification loop is called the “Ping-Pong” amplification pathway [25, 46].

### Characteristics of piRNAs

At present, the relevant databases have estimated that the human genome has nearly 23,000 piRNAs, markedly higher than that of miRNAs (approximately 2000) but similar to that of proteins encoded by mRNA genes (approximately 20,000) [47, 48]. The abundance of piRNAs reveals that piRNAs may play a role in gene regulation, similar to other small ncRNAs.

The differences between piRNAs and other small ncRNAs in humans are shown in Table 1. piRNAs, small interfering RNAs (siRNAs), and miRNAs are three major groups of regulatory small ncRNAs. These groups with different modes of biogenesis and target regulation share some common features, such as their abilities to guide Argonaute proteins to target nucleic acids in a sequence-dependent manner [24]. However, piRNAs have three unique features that distinguish them from their counterparts. First, piRNAs are generated from intergenic regions (known as piRNA clusters) through a Dicer-independent mechanism. Second, unlike siRNAs and miRNAs, which has double-stranded transcriptional precursors, the precursors of piRNAs are single-stranded transcripts. Finally, piRNAs show 2′-O-methyl modification at the 3′-end and specifically bind to PIWI proteins to perform their corresponding function in the human body [49].

**Table 1:** Differences between piRNAs and other small ncRNAs in humans

Characteristics	piRNA	miRNA	siRNA
Length	24–31 nt	18–25 nt	21–23 nt
Small RNA precursors	Single-stranded RNA	Hairpin RNA	Double-stranded RNA/hairpin RNA
3′ end features	2′-O-methylation	No methylation	2′-O-methylation/No methylation
Generation mechanism	Dicer-independent	Dicer-dependent (asymmetric processing)	Dicer-dependent (symmetric processing)
Function	mRNA and transposon repression, DNA methylation, histone modification, protein interaction	mRNA repression	mRNA repression
Associated proteins	PIWI/PIWI-like/AGO/AUB	AGO1-4	AGO1-4
Silencing mechanism	Transcriptional and posttranscriptional	Posttranscriptional	Transcriptional
Major target genes	Transposons and other genes	Coding genes	Transposons and exogenous genes

## Regulatory mechanisms and biological functions of piRNAs

To date, extensive research has elaborated the functions of piRNAs in animal germ cells [2, 50], and the studies show that piRNAs maintain the genomic integrity of germ cells by inhibiting the activity of transposable elements (TEs) [51]. In human genetic diseases, including cancers, the phenomenon of TE silencing has also been reported [52, 53]. Some studies have shown that piRNAs can guide transposon inhibition, recognize a large number of complementary sequences, achieve transcriptional silencing through epigenetic mechanisms [41, 54], and achieve posttranscriptional inhibition via the formation of piRNA/PIWI complexes [36], thereby regulate cancer progression at multiple omics levels.

### piRNA/PIWI complexes mediate target gene silencing at the transcriptome level

**Fig. 2:**
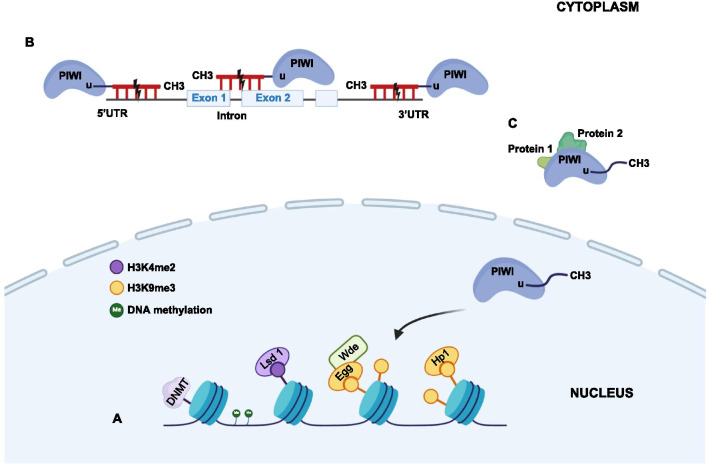
Regulatory mechanisms and biological functions of piRNAs and piRNA/PIWI complexes. (**A**) TGS mainly occurs on PIWI proteins with low catalytic activity. The role of piRNAs in gene expression modification is similar to that of DNA methylation. (**B**) PTGS usually requires PIWI proteins to perform cleavage because it depends on target transcriptional fragments. Here, piRNAs play a role similar to that of miRNAs. (**C**) Both piRNAs and PAZ domains of PIWI proteins in the piRNA/PIWI complex can directly bind to some proteins and thus promote multiprotein interactions

Given that many different PIWI proteins and effectors bind to piRNAs and that the target regulation patterns of piRNA/PIWI complexes are also diverse, there are two mechanisms through which piRNA/PIWI complexes regulate gene transcription. One is transcriptional gene silencing (TGS), which mainly occurs with PIWI proteins with low catalytic activity (Fig. 2A) [55, 56]. In TGS, the piRNA/PIWI complex mediates chromatin silencing by collaborating with histone-modifying enzymes or DNA methyltransferases and thereby suppresses mRNA transcription [2, 57, 58]. Epigenetic modification has a special role in tumorigenesis, and ncRNAs, particularly young piRNAs, play an important role in epigenetic modification [59]. A recent study found that in mouse fetal gonocytes, SPOCD1, a nuclear protein expressed only during the de novo genomic methylation period that acts as a Miwi2-related factor, binds Miwi2 to the transcripts of nascent TEs and recruits suppressive chromatin remodeling activities and de novo methylation devices [60]. Zhang et al. [9] elucidated that the piR-31470/PIWIL4 complex could regulate the methylation level of *GSTP1* by recruiting multiple DNA methylation enzymes, thus promote prostate cancer progression. Moreover, in multiple myeloma cells, piRNA-823 could cause aberrant DNA hypermethylation and induce carcinogenesis by DNMT3B activation [61, 62]. In esophageal squamous cell carcinoma (ESCC) tissues, upregulated piR-823 may play a tumorigenic role by inducing abnormal DNA methylation of DNMT3B through epigenetic inheritance [63].

Another mechanism is posttranscriptional gene silencing (PTGS), in which PIWI proteins are usually needed to perform cleavage because PTGS depends on target transcriptional fragments (Fig. 2B) [55, 64, 65]. piRNAs regulate posttranscriptional networks by interacting with RNAs, including mRNAs [66], transcribed pseudogenes [22], and lncRNAs [67], and play a role similar to that of miRNAs. Peng et al. [66] found that piR-55490 could bind to the 3′UTR of *mTOR*, induce mRNA degradation, and thus promote lung cancer progression.

### piRNA/PIWI complexes interact with proteins at the proteome level

Both piRNAs and the PAZ domains of PIWI proteins in the piRNA/PIWI complex can directly bind to some proteins and promote multiprotein interactions (Fig. 2C). For example, piR-823, which inhibits heat shock protein (HSP) expression, binds to HSF1, a common transcription factor of HSPs, and thus promotes phosphorylation at Ser326 in colorectal cancer [68]. Another study revealed that the piR-54265/PIWIL2 complex could recruit STAT3 and P-SRC via the PAZ domain of PIWIL2, form the PIWIL2/STAT3/P-SRC complex, promote STAT3 phosphorylation and signaling pathway activation, and finally contribute to colorectal tumorigenesis [19].

## piRNAs related to cancer hallmarks

**Fig. 3:**
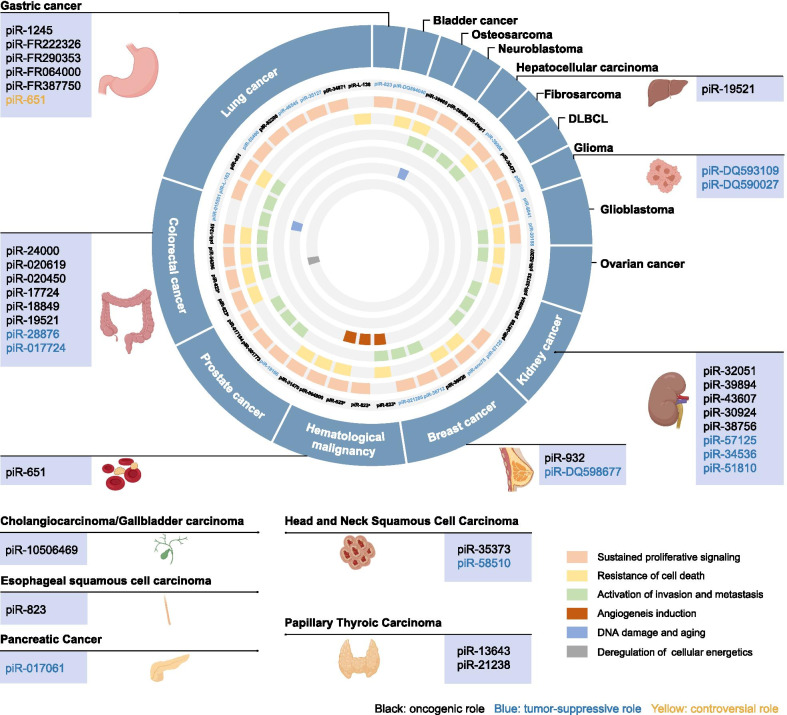
piRNAs involved in human cancers. piRNAs related to cancer hallmarks are shown in the circle. The other five tumor hallmarks (genome instability and mutation, avoidance of immune destruction, evasion of growth suppression, acquisition of replicative immortality, and tumor-promoting inflammation) were not found to involve piRNAs. Other aberrant piRNAs in human cancers are shown in the box. The piRNAs shown in black have an oncogenic role, the piRNAs in blue have a tumor-suppressive role, and those shown in yellow have reportedly controversial roles in the specific cancer type. Abbreviations: DLBCL, diffuse large B-cell lymphoma. ^a^Different studies of piR-823 in specific cancer types

To date, dozens of aberrantly expressed piRNAs have been identified to be involved in the occurrence and progression of cancer (Fig. 3). Their functions and mechanisms can be summarized in terms of several representative hallmarks of cancer. The cancer hallmarks summarize the carcinogenic process behind the main phenotypic changes observed in cancer [69]. These changes overcome the original anticancer defense mechanisms in cells and tissues. In 2011, the originally identified hallmark changes were updated to mainly include sustained proliferative signaling, avoidance of immune destruction, tumor-promoting inflammation, angiogenesis induction, evasion of growth suppressors, acquisition of replicative immortality, activation of invasion and metastasis, resistance of cell death, and deregulation of cellular energetics [70]. For example, the well-known piRNA piR-823 affects the progression of colorectal cancer, gastric cancer, ESCC, and hematological malignancy through multiple pathways, such as tumor cell proliferation, apoptosis, angiogenesis, and energy metabolism [14, 61, 63]. To elucidate the specific mechanisms through which piRNAs lead to tumorigenesis, we classified the reported piRNAs according to their role in cancer hallmarks (Fig. 3) and elaborated their functions in detail.

### Sustained proliferative signaling

Most studies suggest that abnormal piRNA expression may result in the uncontrolled proliferation of cancer cells [11, 12, 14, 61, 68, 71–76]. Tumor cells deregulate chronic proliferative signals and then activate classic signaling pathways, such as the *PI3K/AKT/mTOR* signaling pathway, to regulate cell cycle progression and cell growth to meet the needs of unlimited cell replication and proliferation [77, 78]. Hence, the expression of piR-004800 has been found to be regulated by the sphingosine-1-phosphate receptor and thus activates or inhibits the *PI3K/AKT/mTOR* signaling pathway [79]. The cooperation of downregulated piR-004800 and the *PI3K/AKT/mTOR* signaling pathway restrains cellular growth in exosomes from bone marrow supernatants of patients with multiple myeloma. Moreover, in multiple myeloma cell lines, the silencing of piR-004800 induces apoptosis in vitro and in vivo. Small RNA transcriptome sequencing revealed that the expression of piR-Hep1 is upregulated in 46.6% of hepatocellular carcinoma tumors compared with adjacent non-tumoral liver tissue [80]. The silencing of piR-Hep1 using a locked nucleic acid (LNA) inhibitor suppresses cell viability and motility. Mechanistically, piR-Hep1 interacts with PIWIL2 to form the piR-Hep1/PIWIL2 complex, and this complex activates the PI3K/AKT pathway and regulates cellular proliferation, survival, and metabolism via protein phosphorylation. In lung cancer, Peng et al. [66] observed that piR-55490 is downregulated in lung carcinoma tissues and cells. Intriguingly, silenced piR-55490 promotes the growth of lung cancer by binding to the *mTOR* 3′UTR and activating the AKT/mTOR pathway. The overexpression of piR-55490 in a lung carcinoma xenograft model results in the inhibition of tumor growth. Additionally, one study found that piR-36712 may affect breast cancer cell proliferation but not apoptosis by suppressing the selenoprotein W1 (*SEPW1*) levels through its pseudogene *SEPW1P* in either MCF-7 or ZR75-1 cells [22]. In colorectal cancer, Weng et al. [18] showed that the downregulation of piR-1245 is indispensable for activation of the P53 pathway. After the silencing of piR-1245 in HCT116 and SW480 cells, a particular effect has been observed on the suppression of proliferation and the induction of apoptosis. All piRNAs confirmed to be capable of regulating cellular proliferation in cancer are shown in Fig. 3.

### Resistance to cell death or apoptosis

Apoptosis is one form of cell death. It is critical to explore the biological regulatory mechanisms in the process of tumor apoptosis regarding the malignant development, metastasis, and drug resistance of tumors [81, 82]. In addition, small molecules associated with the apoptotic pathway have been employed in cancer therapies [83, 84]. Using a piRNA molecular beacon, a previous study revealed that the upregulation of piR-36026 inhibits the gene expression of *SERPINA1* and *LRAT*, which are known for their tumor-suppressing functions, and then promotes cell proliferation and inhibits apoptosis in breast cancer [85]. He et al. [34] found that the overexpression of snoRNA-derived piRNA piR-sno75 in breast cancer cells could upregulate TRAIL, a classic apoptotic protein. Mechanistically, piR-sno75 recruits the MLL3/compass-like complex to induce H3K4 methylation and H3K27 demethylation in the promoter region of TRAIL and triggers exogenous apoptosis pathways by interacting with DR4 and DR5. Chu et al. utilized piRNA microarray technology to determine the overall expression of piRNAs in three groups of bladder cancer and adjacent tissues. The results indicated that the piRNA DQ594040 is downregulated in bladder cancer and that the overexpression of DQ594040 could inhibit the proliferation and reduce the apoptosis of bladder cancer cells [86]. The downregulation of piR-39980 significantly attenuates apoptosis of the HT1080 fibrosarcoma cell line and functions as a tumor suppressor [87]. Through a flow cytometric cell cycle analysis, DAPI staining, and acridine orange/ethidium bromide (AO/EB) dual staining, the authors also found that piR-39980 induces proliferation in fibrosarcoma cells. Conversely, piR-39980 is upregulated in neuroblastoma and osteosarcoma cell lines, and the transient overexpression of piR-39980 plays an oncogenic role and promotes tumor progression without affecting the classical apoptosis pathway [20, 88].

### Activation of invasion and metastasis

The enhancement of tumor cell invasion and migration is closely relevant to the malignant progression of tumors and is also an important process in tumor development. Three piRNAs (piR-57125, piR-38756, and piR-30924) exhibit a strong association with tumor metastasis in genome-wide microarray and RT-qPCR analyses of clear cell renal cell carcinomas [89]. In prostate cancer, piR-001773 and piR-017184 synergistically regulate the transcription of protocadherin 9 (*PCDH9*) under homeostasis and thereby promote tumor invasion and metastasis [90]. Another study showed that the cooperation of PIWIL3, piR-30188, OIP5-AS1, and miR-367-3p regulates the migration and invasion of glioma cells. Their target gene, *CEBPA*, regulates TNF receptor-associated factor 4 and PIWIL3 and forms a feedback loop to control cellular growth [67].

### Angiogenesis induction

In general, tumor angiogenesis strengthens the interaction between blood vessels and tumor cells to lead to tumor growth and metastasis [91]. Few studies have investigated the role of piRNAs in promoting tumor angiogenesis, and most of these have focused on piRNA-823 [61, 62, 72]. In multiple myeloma, piRNA-823 may promote tumorigenesis by regulating de novo DNA methylation and angiogenesis [61]. Mechanistically, piRNA-823 induces a decrease in VEGF secretion to reduce proangiogenic activity among multiple myeloma cells. The VEGF pathway could play a neoangiogenic function in multiple myeloma cells in a paracrine manner and thus promotes the occurrence of tumors. Another mechanistic study showed that granulocytic myeloid-derived suppressor cells (G-MDSCs) induce piRNA-823 expression to enhance the tumorigenicity of multiple myeloma cells through DNA methylation [62]. In addition, the inhibition of piRNA-823 in multiple myeloma cells decreases the stemness of multiple myeloma stem cells preserved by G-MDSCs and reduces the tumor burden and angiogenesis in vivo. Li et al. [72] revealed that piRNA-823 delivered by multiple myeloma-derived extracellular vesicles contributes to the invasion of endothelial cells by regulating the *ICAM-1* and *CXCR4* levels.

### Deregulation of cellular energetics

The main feature of tumors is the rapid proliferation of cells. During the process of rapid proliferation, energy metabolism is needed to promote cell growth and division [70]. Therefore, excessive energy and metabolic reprogramming, which are regulated by various carcinogens and tumor suppressors, are important parts of human cancer that differentiate cancer from normal tissues [92]. For example, Feng et al. [14] found that upregulated piRNA-823 in colorectal cancer cells could inhibit the ubiquitination of hypoxia-inducible factor-1 (*HIF-1*) by increasing the expression of glucose-6-phosphate dehydrogenase (*G6PD*), and this inhibition increases glucose consumption in cancer cells and reduces the intracellular reactive oxygen species content.

### DNA damage and aging

DNA damage is a permanent change in DNA nucleotide sequence that occurs during replication. DNA sequence polymorphisms caused by a single nucleotide mutation at the genome level are called single nucleotide polymorphisms (SNPs), and these SNPs may also lead to DNA damage. SNPs increase the susceptibility to cancer mainly through two routes. On the one hand, SNPs in piRNA biogenesis pathway-related genes may alter the process of piRNA production, affect piRNA maturation and expression [93, 94]. Genes that have an important influence on the process of piRNA production are called piRNA biogenesis pathway-related genes. The core effector complex of the piRNA biogenesis pathway is mainly composed of piRNAs and proteins in the PIWIL protein subfamily (HIWI/PIWIL1, HILI/PIWIL2, HIWI3/PIWIL3, and HIWI2/PIWIL4) [95]. The PIWI protein family and related factors play a vital role in various cellular processes and diseases, and the genetic variations that occur in these genes have the potential to regulate the function of piRNAs. Zhang et al. [96] found that the tag SNP rs11551405 in *DCP1A*, which is a piRNA biogenesis pathway-related gene, is associated with an increased risk of melanoma-specific death. On the other hand, SNPs may not change the maturation and expression of piRNA, but affect the interaction of piRNA with other RNAs or proteins, thus affect the risk of tumor development. Another study revealed that the SNP rs11776042 may be involved in the generation of piR-015551 from LNC00964-3, which is significantly associated with a decreased risk of colorectal cancer [97].

Additionally, aggregation of DNA damage causes aging, making normal cells less efficient and inevitably leading to disease or cancer [98]. The regulation of TEs by piRNAs can affect DNA damage through aging speed [99]. A study showed that piR-39980 could induce neuroblastoma cellular senescence by changing the regulation of JAK3 expression through a route independent of classical apoptosis pathways [20].

Overall, the discovery of small ncRNAs has led to profound alterations in our perceptions of the mechanisms of gene control in cancer. To date, most of the functional studies on piRNAs have focused on the effects on tumor cell characteristics, such as the effects on tumor cell proliferation, apoptosis, invasion, and metastasis. However, these only scratch the surface of the real complexity, and the detailed mechanisms in tumors have not been thoroughly studied. In particular, whether piRNAs can affect other cancer hallmarks, such as avoidance of immune destruction or tumor-promoting inflammation, is worth exploring in the future.

## piRNAs as diagnostic and prognostic biomarkers in cancer

Tumor screening is an important approach for the early detection of cancer and precancerous lesions [100–102]. Small RNAs such as miRNAs have received abundant attention as potential noninvasive biomarkers for early cancer screening [103]. piRNAs can also be used as biomarkers for cancer screening. For example, piR-54265 is a molecular marker for early colorectal cancer screening in the general population [104]. Additionally, the study of clinical prognostic indicators is valuable for improving the life quality of patients with cancer. Most studies have focused on the application of piRNAs as diagnosis and prognostic markers for patients with cancer. The clinical applications of piRNAs as potential diagnostic and prognostic biomarkers in a variety of cancers are shown in Table 2.

**Table 2:** Clinical applications of piRNAs in cancer

piRNA	Cancer	Sample type	Expression in tumors	Diagnosis biomarker	Prognostic biomarkers	Chemoresistance	Therapeutic targets	Clinical relevance	References
piR-36712	Breast cancer	Tissues	↓	√	√	√	√	Metastatic	[22]
piR-932		Cell lines	↑					–	[108]
piR-4987		Tissues	↑	√				Metastatic	[113]
piR-20365		Tissues	↑	√				–	[113]
piR-20485		Tissues	↑	√				–	[113]
piR-20582		Tissues	↑	√				–	[113]
piR-sno75		Cell lines/tissues	↓			√	√	–	[34]
piR-10506469	CHOL	Plasma exosomes	↑	√				–	[105]
piR-24000	Colorectal Cancer	Tissues	↑	√				Metastatic, differentiation, tumor stage	[13]
piR-020619		Serum	↑	√				–	[107]
piR-020450		Serum	↑	√				–	[107]
piR-54265		Serum	↑	√			√	Tumor stage, relapse	[104]
piR-823		Tissues	↑		√		√	Metastatic, tumor stage, Adjuvant chemotherapy	[14, 68]
piR-017724		Serum	↓				√	–	[109]
piR-18849		Tissues	↑		√			Metastatic, differentiation	[17]
piR-19521		Tissues	↑		√			Metastatic, differentiation	[17]
piR-1245		Tissues	↑		√			Metastatic	[18]
piR-54265		Tissues	↑		√	√	√	–	[19]
piR-5937		Serum	↓	√				–	[114]
piR-28876		Serum	↓	√				–	[114]
piR-30473	DLBCL	Tissues	↑		√			–	[10]
piR-823	ESCC	Tissues	↑	√	√			Metastatic	[63]
piR-10506469	Gallbladder carcinoma	Plasma exosomes	↑	√				Tumor stage	[105]
piR-1245	Gastric cancer	Gastric juice	↑	√				Tumor stage, tumor size	[15]
piR-823		Tissues	↓				√	–	[71]
piR-651		Tissues	↑	√			√	–	[16]
piR-8041	Glioblastoma	Tissues	↓	√			√	–	[112]
piR-30188		Tissues	↓	√				–	[67]
piR-DQ593109	Glioma	Cell lines	↑				√	–	[118]
piR-651	Hematological malignancy	Serum	↑			√		–	[21]
piR-823		Cell lines	↑					Tumor stage	[61]
piR-823		Cell lines	↑				√	Tumor stage	[72]
piR-004800		Cell lines/exosomes	↑				√	–	[79]
piR-34736	HNSCC	Tissues	↑	√				–	[116]
piR-34536	Kidney cancer	Tissues/serum	↓		√			–	[110]
piR-51810		Tissues/serum	↓		√			Metastatic	[110]
piR-32051		Tissues	↑	√				–	[7]
piR-43607		Tissues	↑		√			–	[7]
piR-39894		Tissues	↑	√				Metastatic	[7]
piR-57125		Tissues	↓		√			Relapse	[89]
piR-30924		Tissues	↑		√			Relapse	[89]
piR-38756		Tissues	↑		√			–	[89]
piR-823		Tissues	↓		√			–	[115]
piR-651	Lung cancer	Tissues	↑	√			√	Tumor stage, relapse	[11, 12]
piR-55490		Tissues	↓		√			–	[66]
piR-L-138		Cell lines	↑			√			[111]
piR-39980	Neuroblastoma	Cell lines	↑			√		–	[20]
piR-017061	pancreatic cancer	Tissues	↓	√				–	[117]
piR-21238	PTC	Tissues	↑	√				Tumor stage	[106]
piR-13643		Tissues	↑	√				Tumor stage	[106]

*CHOL* cholangiocarcinoma, *DLBCL* diffuse large B-cell lymphoma, *ESCC* esophageal squamous cell carcinoma, *HNSCC* head and neck squamous cell carcinoma, *PTC* papillary thyroid carcinoma

In both patients with cholangiocarcinoma and patients with gallbladder carcinoma, the expression of piR-10506469 in blood one week after surgery is significantly decreased compared with that before surgery [105]. Similarly, the level of piR-10506469 in patient plasma exosomes is significantly increased compared with that in healthy individuals, which suggests its unique diagnostic value. In ESCC, piR-823 is positively correlated with *DNMT3B* expression [63]. The authors revealed that the carcinogenic effect of piR-823 may result from its induced abnormal DNA methylation via *DNMT3B*. Additionally, an increased risk of lymph node metastasis is associated with a higher piR-823 level, and piR-823 exhibits high specificity for detecting ESCC (AUC = 0.713), which suggests its potential as a diagnostic and prognostic biomarker. In NSCLC, piR-651 can induce tumor progression by affecting the *cyclin D1*/*CDK4* pathway and acts as a potential diagnostic indicator [11, 12]. Using piRNA sequencing data from papillary thyroid carcinoma (PTC), researchers have identified two upregulated piRNAs, piR-13643 and piR-21238 and found that these two piRNAs are associated with clinical stages and exhibit better specificity for distinguishing between benign and malignant nodules than the current biomarker *HBME1* [106].

Interestingly, the expression of piR-1245 in gastric juice is higher in patients than in control individuals, with an AUC value of 0.885 [15]. Moreover, the expression of piR-1245 in gastric juice has the potential to be a noninvasive biomarker for gastric cancer detection and prognosis. In another study, the well-known piRNA piR-651 was found to be a potential marker for gastric cancer diagnosis [16]. In colorectal cancer, piR-24000 acts as a possible oncogene that can significantly distinguish patients with colorectal cancer from normal individuals [13]. Additionally, serum piR-54265, piR-020619, and piR-020450 were also found to serve as sensitive and specific noninvasive biomarkers for early colorectal cancer detection in previous studies [104, 107]. Another research group used a piRNA microarray to identify a well-known oncogenic piRNA, piRNA-823, which is highly expressed in colorectal cancer tissues [14]. In this study, the authors performed multivariate analysis and found that piRNA-823 is an independent predictor of overall survival, which suggests its important role in colorectal cancer carcinogenesis. Furthermore, piR-19521, piR-18849, and piR-1245 also show novel independent prognostic characteristics and have the potential to be used as prognostic markers in colorectal cancer [17, 18].

In addition to the above-mentioned piRNAs, piRNAs that have been clinically correlated with breast cancer, lung cancer, kidney cancer, and glioblastoma are shown in Table 2 [34, 108–118]. Given the activation of piRNAs in germline cells, abnormal expression of piRNAs is more sensitive to the diagnosis of testicular cell tumors compared with that of other small ncRNAs [119]. Moreover, the expression of piRNAs in circulation is relatively stable and is easily detected in body fluids, such as blood, serum, or gastric juice. Thus, piRNAs may have great potential for early non-invasive cancer screening in the general population and diagnosis in patients with cancer, but there still remain some disadvantages to the efficiency of piRNAs. First, some piRNA tests using patient’s tissues, which can be harmful to patients, are still needed for diagnosis. Second, compared with the existing tests, the detection and identification of piRNAs in tissue or body fluids are more expensive, which restricts the extensive use of piRNAs as biomarkers. Third, the reliability of using piRNAs for diagnosis still needs to be proven.

## piRNAs and cancer therapy resistance

Cancer therapies mainly include radiotherapy and chemotherapy. The combination of targeted therapy and chemotherapy significantly prolongs the overall survival of patients with advanced disease and improves their quality of life [120]. However, primary or acquired drug resistance ultimately results in therapeutic failure and poor prognosis in cancer patients [121]. Therefore, the development of new strategies is urgently needed to improve therapeutic responses. Many mechanisms, including reduced drug accumulation, enhanced DNA repair, a favorable tumor microenvironment (TME) and target gene amplification, have been proven to be involved in therapy resistance, and piRNAs may be a novel therapeutic target for human cancer [122–124]. In recent years, the role of multiple ncRNAs in regulating metastasis and recurrence of malignancies has received increased attention [14, 125–128]. Nevertheless, studies of piRNAs in cancer therapy, particularly in cancer resistance to radiotherapy or chemotherapy, are still at the nascent stage. The specific mechanism through which piRNAs affect resistance is related to chemotherapy-induced decreases in apoptosis, which makes tumor cells insensitive. This section highlights the functions of piRNAs in modulating therapy resistance in different cancers and discusses the limitations of the available knowledge and future potential directions.

In detail, piR-39980 has been identified as a key contributor to chemoresistance to doxorubicin in neuroblastoma cells and inhibits drug-induced apoptosis [20]. In the study, the researchers transfected IMR-32 cells with piR-39980 at different concentrations of doxorubicin and then measured the viability of the cells. Compared with cells treated with a single drug, cells overexpressed piR-39980 exhibited reduced sensitivity to doxorubicin, which might have led to the development of drug resistance. Another study examined the serum piR-651 levels in 94 patients with classical Hodgkin lymphoma and found that lower levels of piR-651 are related to increased overall and disease-free survival and complete response to first-line treatment [21]. Tan et al. [22] revealed that the overexpressed piR-36712 in ZR75-1 and MCF7 cells markedly reduces the IC_50_ values of doxorubicin and paclitaxel in these cells, whereas the inhibition of piR-36712 has the opposite effect, and these patterns have been confirmed in mouse xenograft models. This synergistic anticancer effect suggests that piR-36712 may be an effective agent for breast cancer treatment. Interestingly, Mai et al. [104] also found a synergistic anticancer effect of piRNAs in colorectal cancer. These authors found that the reduced apoptosis induced by chemotherapy drugs results in the insensitivity of piR-54265-overexpressed colorectal cancer cells to chemotherapy drugs. Similarly, studies using a mouse xenograft tumor model using oxaliplatin, 5-FU or a combination regimen found that subcutaneous tumors formed by colorectal cancer cells overexpressed piR-54265 exhibit increased resistance to chemotherapy, whereas subcutaneous tumors with downregulated piRNA expression show an excellent response to chemotherapy. These results suggest that targeting piR-54265 may provide a novel approach for colorectal cancer treatment.

## Databases for piRNAs and functional predictions

As high-throughput sequencing technology improves, many programs have been developed to identify piRNAs from small RNA data, and these include Piano, piRNAPredictor, piRPred, 2L-piRNA, Pibomd, IpiRId, V-ELMpiRNAPred, proTRAC, piClust, and piRNN [129–138]. For example, piRPred is a machine learning method that uses support vector machine classifiers and multiple kernels to identify piRNAs based on four unique sequence features of piRNAs [131]. In non-model organisms, the deep learning model developed by piRNN using the convolution neural network framework exhibits better performance [138]. Subsequently, some available databases have integrated different types of piRNA data to assist the functional research of piRNAs (Table 3). However, due to the different naming rules and numbers of piRNA in each database, the names of piRNAs in various studies are not consistent, thus a unified naming standard should be established for the study of piRNAs. For example, hsa-piR-30025 and hsa_piRNA_30025 are listed in the piRNAdb and piRNAQuest databases, respectively, but have different chromosomal positions. In addition, there is no uniform naming strategy for piRNAs in the National Center for Biotechnology Information (NCBI) GenBank, and various data sources are included in this database.

**Table 3:** Details of databases for piRNAs and functional predictions

Databases	Characteristics	Organisms	Website
piRBase	This database focuses on the comprehensive annotation of piRNA sequences and contains potential piRNA targets and disease-related piRNAs	21 organisms^a)^	http://www.regulatoryrna.org/database/piRNA/
RNAdb 2.0	This database contains over 88,000 piRNA candidates that have been cloned and sequenced from mice, humans, and rats	Human, mouse and rat	http://research.imb.uq.edu.au/RNAdb/
piRNA Cluster Database	This database provides comprehensive data on piRNA clusters in multiple species, tissues, and developmental stages based on small RNA sequence data deposited in NCBI’s Sequence Read Archive (SRA)	51 organisms^b)^	https://www.smallrnagroup.uni-mainz.de/piCdb/
piRNAdb	This database is a PIWI-interacting RNA sequence storage and search system that provides some other types of relevant information such as alignments, clusters, datasets, and targets of piRNAs	6 organisms^c)^	https://www.pirnadb.org/
piRNABank	This database compiles all the possible clusters of piRNAs and depicts piRNAs along with the associated genomic elements such as genes and repeats on a genome-wide map	Human, mouse, rat, and Drosophila	http://pirnabank.ibab.ac.in/
piRNAQuest	This database provides annotation of piRNAs based on their genomic location in gene, intron, intergenic, CDS, UTR, repeat element, pseudogene, and syntenic regions. The database also contains information on all possible piRNA clusters along with significant motifs present within the piRNAs comprising a cluster	Human, mouse and rat	http://bicresources.jcbose.ac.in/zhumur/pirnaquest/
IsopiRBank	This database stores piRNA isoforms detected from small RNA sequencing data, performs detailed classification, and integrates annotation information as well as genome mapping results. Furthermore, target analysis and enrichment analysis revealing the piRNA isoform roles in certain biological processes are included	Human, mouse, Drosophila, and zebrafish	http://mcg.ustc.edu.cn/bsc/isopir/
piRNA-eQTL	This database demonstrates the effects of genetic variation on piRNA expression based on the Cancer Genome Atlas (TCGA) project and is a user-friendly database for the analysis of cis-piRNA eQTLs for 33 cancer types	Human	http://njmu-edu.cn:3838/piRNA-eQTL/
pirScan	This database allows C. elegans researchers to predict piRNA-targeting sites and to avoid the persistent germline silencing of transgenes that has rendered many constructs unusable	C. elegans or C. briggsae	http://cosbi4.ee.ncku.edu.tw/pirScan/
piRTarBase	This database allows researchers to explore piRNA-targeting sites and their regulatory effects on endogenous genes and identify predicted mRNA targets of specific piRNAs	C. elegans or C. briggsae	http://cosbi6.ee.ncku.edu.tw/piRTarBase/

^a)^21 organisms: C. elegans, chicken, cow, crab-eating macaque, D. erecta, D. melanogaster, D. virilis, D. yakuba, human, marmoset, mouse, pig, rabbit, rat, rhesus, sea hare, silkworm, starlet sea anemone, tree shrew, X. tropicalis, and zebrafish

^b)^51 organisms: African clawed frog, African malaria mosquito, American alligator, Arizona bark scorpion, big brown bat, blue-eyed rice fish, brown rat, budgerigar, cabbage looper, Carolina anole, cattle (col. cow), common house spider, common marmoset, crab-eating macaque, diamondback moth, European honey bee, European rabbit, fruit fly, giant tiger prawn, goldfish, gray mouse lemur, gray short-tailed opossum, great pond snail, Herbst’s burying beetle, horse, house mouse, housefly, human, Japanese rice fish, large earth bumblebee, large milkweed bug, migratory locust, northern tree shrew, oriental fruit fly, Pacific oyster, pea aphid, pig, platypus, red flour beetle, rhesus macaque, speckled wood, three-spined stickleback, vinegar fly, West African fruit fly, western clawed frog, wolf (dog), yakuba fruit fly, yellow fever mosquito, yellowhead catfish, and zebrafish

^c)^Six organisms: Caenorhabditis elegans, Cricetus griseus, Drosophila melanogaster, human, Mus musculus, and Rattus norvegicus

At present, piRBase is the largest and most complete piRNA database, with 264 datasets from 21 species [139, 140], and it is an expert database in RNAcentral. Since piRBase was launched by He et al. in 2014, researchers have systematically discovered the shearing and regulatory effect of piRNAs on coding genes [44], developed a piRNA target gene prediction algorithm [141], and upgraded the database in 2018. Given the functional diversity of piRNAs, piRBase divides piRNAs into two categories according to their biogenesis: repeat-derived and gene-overlapping piRNAs. In addition, piRBase also includes epigenetic data and predicted lncRNAs and gene targets of piRNAs. Notably, the piRNA and cancer data in piRBase cover eight types of cancer (breast, bladder, pancreatic, gastric, liver, kidney, colorectal cancer, and myeloma) to assist cancer-related studies.

Another database, RNAdb 2.0, also stores more than 88,000 candidate piRNAs from mice, humans, and rats [142]. One study compared piRNA sequences with the sequences of other ncRNAs using the RNAdb 2.0 and piRNABase databases and showed that piRNAs in human plasma consist of ncRNA fragments that are the same as those in human tumor tissues [143]. The piRNA Cluster Database provides comprehensive data on piRNA clusters based on tissues from 12 species and developmental stages [144]. piRNAdb, which was developed by The Bioinformatics Laboratory at the Molecular Oncology Center in Brazil, provides other relevant information, such as alignments, clusters, datasets, and targets of piRNAs.

Additionally, piRNABank, originally founded by Lakshmi et al. [47], is a network resource with classified and clustered piRNAs. The database is a user-friendly resource that stores comprehensive information about piRNAs from humans, mice, rats, and Drosophila. It includes all possible piRNA clusters and supports a comprehensive search function for organisms and chromosomes, including accession numbers, chromosomal locations, gene names or symbols, sequence homology-based searches, clusters, and corresponding genes and repeating elements. Another database, piRNAQuest, also contains information about all possible piRNA clusters [145]. Detailed classifications of piRNA isoforms detected by small RNA sequencing data are included in IsopiRBank [146]. This database can be used to reveal the role of piRNA isomers in some biological processes through target analysis and enrichment analysis. The other two databases, pirScan and piRTarBase, contain piRNA information from Caenorhabditis elegans or Caenorhabditis briggsae [147, 148]. Researchers have been able to predict piRNA target sites and identify expected mRNA targets of piRNAs.

In addition, our research group recently constructed a piRNA-expression quantitative trait locus (eQTL) database to reveal the effects of SNPs and piRNAs on the genetic mechanism of cancer at the genomic level [149]. Based on the genotyping and piRNA expression data of 33 cancer types in The Cancer Genome Atlas (TCGA), millions of SNP-piRNA pairs have been identified via eQTL analysis in both tumor tissues and normal tissues. This database provides the first combination of genetic variation with piRNAs, which will generate new ideas for further research on cancer etiology.

## Conclusions and perspectives

Abundant studies have reported the important role of ncRNAs in the regulation of different types of cancer [150, 151]. Compared with lncRNAs, miRNAs, and circRNAs, piRNAs are a relatively new type of ncRNA and have attracted increasing attentions in recent years. Due to the discovery that piRNAs are related to many types of tumors, their mechanisms and functions need to be explored. Although the expression of piRNAs can be easily detected through the development of next-generation sequencing technology, there remain many unknown piRNAs due to the complicated mechanisms through which they are generated, the difficulty in their identification, the lack of universal rules for piRNA naming, the instability of the detection results, and the current lack of knowledge on their regulatory mechanisms. Several limitations and perspectives are illustrated below.

As mentioned above, the three main regulatory mechanisms and biological functions of piRNAs are TGS, PTGS, and protein–protein interactions. In TGS, piRNAs appear to play a role in gene expression modification that is similar to DNA methylation. In general, piRNAs mainly affect tumor development by regulating DNA methylation transferase and then cause aberrant DNA hypermethylation. However, whether DNA methylation could reversely affect the expression or functions of piRNAs remains unclear and deserves further discussion. Moreover, the dual mechanism of the piRNA/MIWI complex in the process of mouse spermiogenesis, which involve inducing the degradation or translational activation of target mRNA via recruitment of related factors, has been investigated [152, 153]. Similarly, whether the piRNA/PIWI complex functions through a similar dual mechanism in human tumor cells is worth investigating. Furthermore, given the base-pairing principle, the variation of a single base in the piRNA-mRNA binding domain may also disrupt piRNA function. Therefore, the effect of genetic variations in the pairing region should be sufficiently explored to identify new piRNA mechanisms in the future.

We also found that most previous studies only described the one-to-one, multiple-to-one, or one-to-multiple relationship between piRNAs and their target genes in tumors, whereas few studies have explored the combined effects of multiple piRNAs in a pan-cancer analysis. In addition, different piRNAs may perform additive or opposite functions. Therefore, the combined effects of multiple piRNAs on multiple targets in tumors should also be addressed in future studies, and the results will contribute to a more comprehensive understanding of the role of piRNAs in the pathogenesis and progression of tumors.

In addition, most of the functions of piRNAs studied to date were limited to their effects on tumor phenotypes. In the past few decades, there has been a substantial increase in the study of the mechanisms of cancer immunity. Similarly, most of the prognostic studies performed to date have focused on the effect on tumor chemotherapy resistance rather than radiotherapy or immunotherapy resistance. Whether piRNAs are involved in the individual immune processes of cancer remains unclear. The role of piRNAs in resistance to immune checkpoint blockade agents in cancers has not been investigated. Therefore, additional studies are needed to better understand the piRNA-mediated regulation of cancer immunotherapy, and the results will lead to the development of novel effective therapeutic strategies for cancer therapy.

Several recent studies demonstrated that PIWI proteins highly expressed in germ cells can still be activated in human tumor cells in the absence of piRNA induction and promote tumorigenesis in different modes [154, 155]. Consequently, these studies have enriched the knowledge of the mechanism of piRNAs and PIWI proteins in the field of human cancer. All of this information makes it possible to induce or exogenously add piRNA as a potential therapeutic strategy for human cancer with high PIWI protein expression in the future.

In short, unlike other ncRNAs, piRNAs, as a relatively newly discovered type of small ncRNA, have rarely been the topic of preclinical studies or clinical trials. In the future, large cohort clinical studies are needed to further explore and verify the potential and advantages of piRNAs as biomarkers for tumor diagnosis, prognosis and therapeutic efficacy. Although there remain many unknown challenges, advances in multiomics, sequencing and other technologies will lead to a more in-depth and comprehensive assessment of piRNAs as a new target for tumor diagnosis and treatment.

## Data Availability

Not applicable.

## Abbreviations

ncRNA: Non-coding RNA
piRNAs: PIWI-interacting RNAs
3′UTRs: 3′ Untranslated regions
TEs: Transposable elements
TGS: Transcriptional gene silencing
ESCC: Esophageal squamous cell carcinoma
PTGS: Posttranscriptional gene silencing
HSP: Heat shock protein
LNA: Locked nucleic acid
SEPW1: Suppressing selenoprotein W1
AO/EB: Acridine orange/ethidium bromide
PCDH9: Protocadherin 9
G-MDSCs: Granulocytic myeloid-derived suppressor cell
HIF-1: Hypoxia-inducible factor-1
G6PD: Glucose-6-phosphate dehydrogenase
SNPs: Single nucleotide polymorphisms
PTC: Papillary thyroid carcinoma
TME: Tumor microenvironment
NCBI: National Center for Biotechnology Information
eQTL: Expression quantitative trait locus
TCGA: The Cancer Genome Atlas

## References

[1] Beermann J, Piccoli MT, Viereck J, Thum T (2016). Non-coding RNAs in development and disease: background, mechanisms, and therapeutic approaches. Physiol Rev.

[2] Czech B, Munafo M, Ciabrelli F, Eastwood EL, Fabry MH, Kneuss E (2018). piRNA-guided genome defense: from biogenesis to silencing. Annu Rev Genet.

[3] Girard A, Sachidanandam R, Hannon GJ, Carmell MA (2006). A germline-specific class of small RNAs binds mammalian Piwi proteins. Nature.

[4] Aravin AA, Naumova NM, Tulin AV, Vagin VV, Rozovsky YM, Gvozdev VA (2001). Double-stranded RNA-mediated silencing of genomic tandem repeats and transposable elements in the D melanogaster germline. Curr Biol.

[5] Grimson A, Srivastava M, Fahey B, Woodcroft BJ, Chiang HR, King N (2008). Early origins and evolution of microRNAs and Piwi-interacting RNAs in animals. Nature.

[6] Ozata DM, Gainetdinov I, Zoch A, O'Carroll D, Zamore PD (2019). PIWI-interacting RNAs: small RNAs with big functions. Nat Rev Genet.

[7] Li Y, Wu X, Gao H, Jin JM, Li AX, Kim YS (2015). Piwi-interacting RNAs (piRNAs) are dysregulated in renal cell carcinoma and associated with tumor metastasis and cancer-specific survival. Mol Med.

[8] Qi T, Cao H, Sun H, Feng H, Li N, Wang C (2020). piR-19166 inhibits migration and metastasis through CTTN/MMPs pathway in prostate carcinoma. Aging (Albany NY).

[9] Zhang L, Meng X, Pan C, Qu F, Gan W, Xiang Z (2020). piR-31470 epigenetically suppresses the expression of glutathione S-transferase pi 1 in prostate cancer via DNA methylation. Cell Signal.

[10] Han H, Fan G, Song S, Jiang Y, Qian C, Zhang W (2021). piRNA-30473 contributes to tumorigenesis and poor prognosis by regulating m6A RNA methylation in DLBCL. Blood.

[11] Yao J, Wang YW, Fang BB, Zhang SJ, Cheng BL (2016). piR-651 and its function in 95-D lung cancer cells. Biomed Rep.

[12] Li D, Luo Y, Gao Y, Yang Y, Wang Y, Xu Y (2016). piR-651 promotes tumor formation in non-small cell lung carcinoma through the upregulation of cyclin D1 and CDK4. Int J Mol Med.

[13] Iyer DN, Wan TM, Man JH, Sin RW, Li X, Lo OS (2020). Small RNA profiling of piRNAs in colorectal cancer identifies consistent overexpression of piR-24000 that correlates clinically with an aggressive disease phenotype. Cancers (Basel).

[14] Feng J, Yang M, Wei Q, Song F, Zhang Y, Wang X (2020). Novel evidence for oncogenic piRNA-823 as a promising prognostic biomarker and a potential therapeutic target in colorectal cancer. J Cell Mol Med.

[15] Zhou X, Liu J, Meng A, Zhang L, Wang M, Fan H (2020). Gastric juice piR-1245: a promising prognostic biomarker for gastric cancer. J Clin Lab Anal.

[16] Cheng J, Guo JM, Xiao BX, Miao Y, Jiang Z, Zhou H (2011). piRNA, the new non-coding RNA, is aberrantly expressed in human cancer cells. Clin Chim Acta.

[17] Yin J, Qi W, Ji CG, Zhang DX, Xie XL, Ding Q (2019). Small RNA sequencing revealed aberrant piRNA expression profiles in colorectal cancer. Oncol Rep.

[18] Weng W, Liu N, Toiyama Y, Kusunoki M, Nagasaka T, Fujiwara T (2018). Novel evidence for a PIWI-interacting RNA (piRNA) as an oncogenic mediator of disease progression, and a potential prognostic biomarker in colorectal cancer. Mol Cancer.

[19] Mai D, Ding P, Tan L, Zhang J, Pan Z, Bai R (2018). PIWI-interacting RNA-54265 is oncogenic and a potential therapeutic target in colorectal adenocarcinoma. Theranostics.

[20] Roy J, Das B, Jain N, Mallick B (2020). PIWI-interacting RNA 39980 promotes tumor progression and reduces drug sensitivity in neuroblastoma cells. J Cell Physiol.

[21] Cordeiro A, Navarro A, Gaya A, Diaz-Beya M, Gonzalez-Farre B, Castellano JJ (2016). PiwiRNA-651 as marker of treatment response and survival in classical Hodgkin lymphoma. Oncotarget.

[22] Tan L, Mai D, Zhang B, Jiang X, Zhang J, Bai R (2019). PIWI-interacting RNA-36712 restrains breast cancer progression and chemoresistance by interaction with SEPW1 pseudogene SEPW1P RNA. Mol Cancer.

[23] Parhad SS, Yu T, Zhang G, Rice NP, Weng Z, Theurkauf WE (2020). Adaptive evolution targets a piRNA precursor transcription network. Cell Rep.

[24] Iwasaki YW, Siomi MC, Siomi H (2015). PIWI-interacting RNA: its biogenesis and functions. Annu Rev Biochem.

[25] Thomson T, Lin H (2009). The biogenesis and function of PIWI proteins and piRNAs: progress and prospect. Annu Rev Cell Dev Biol.

[26] Lau NC, Seto AG, Kim J, Kuramochi-Miyagawa S, Nakano T, Bartel DP (2006). Characterization of the piRNA complex from rat testes. Science.

[27] Czech B, Hannon GJ (2016). One loop to rule them all: the ping-pong cycle and piRNA-guided silencing. Trends Biochem Sci.

[28] Jensen S, Brasset E, Parey E, Roest Crollius H, Sharakhov IV, Vaury C (2020). Conserved small nucleotidic elements at the origin of concerted piRNA biogenesis from genes and lncRNAs. Cells.

[29] Krishnan P, Ghosh S, Wang B, Heyns M, Li D, Mackey JR (2016). Genome-wide profiling of transfer RNAs and their role as novel prognostic markers for breast cancer. Sci Rep.

[30] Keam SP, Young PE, McCorkindale AL, Dang TH, Clancy JL, Humphreys DT (2014). The human Piwi protein Hiwi2 associates with tRNA-derived piRNAs in somatic cells. Nucleic Acids Res.

[31] Honda S, Kawamura T, Loher P, Morichika K, Rigoutsos I, Kirino Y (2017). The biogenesis pathway of tRNA-derived piRNAs in Bombyx germ cells. Nucleic Acids Res.

[32] Ha H, Song J, Wang S, Kapusta A, Feschotte C, Chen KC (2014). A comprehensive analysis of piRNAs from adult human testis and their relationship with genes and mobile elements. BMC Genom.

[33] Zhong F, Zhou N, Wu K, Guo Y, Tan W, Zhang H (2015). A SnoRNA-derived piRNA interacts with human interleukin-4 pre-mRNA and induces its decay in nuclear exosomes. Nucleic Acids Res.

[34] He X, Chen X, Zhang X, Duan X, Pan T, Hu Q (2015). An Lnc RNA (GAS5)/SnoRNA-derived piRNA induces activation of TRAIL gene by site-specifically recruiting MLL/COMPASS-like complexes. Nucleic Acids Res.

[35] Scott MS, Ono M, Yamada K, Endo A, Barton GJ, Lamond AI (2012). Human box C/D snoRNA processing conservation across multiple cell types. Nucleic Acids Res.

[36] Liu Y, Dou M, Song X, Dong Y, Liu S, Liu H (2019). The emerging role of the piRNA/piwi complex in cancer. Mol Cancer.

[37] Nishimasu H, Ishizu H, Saito K, Fukuhara S, Kamatani MK, Bonnefond L (2012). Structure and function of Zucchini endoribonuclease in piRNA biogenesis. Nature.

[38] Ipsaro JJ, Haase AD, Knott SR, Joshua-Tor L, Hannon GJ (2012). The structural biochemistry of Zucchini implicates it as a nuclease in piRNA biogenesis. Nature.

[39] Han BW, Wang W, Li C, Weng Z, Zamore PD (2015). Noncoding RNA. piRNA-guided transposon cleavage initiates Zucchini-dependent, phased piRNA production. Science.

[40] Han BW, Zamore PD (2014). piRNAs. Curr Biol.

[41] Luteijn MJ, Ketting RF (2013). PIWI-interacting RNAs: from generation to transgenerational epigenetics. Nat Rev Genet.

[42] Ross RJ, Weiner MM, Lin H (2014). PIWI proteins and PIWI-interacting RNAs in the soma. Nature.

[43] Stein CB, Genzor P, Mitra S, Elchert AR, Ipsaro JJ, Benner L (2019). Decoding the 5' nucleotide bias of PIWI-interacting RNAs. Nat Commun.

[44] Zhang P, Kang JY, Gou LT, Wang J, Xue Y, Skogerboe G (2015). MIWI and piRNA-mediated cleavage of messenger RNAs in mouse testes. Cell Res.

[45] Webster A, Li S, Hur JK, Wachsmuth M, Bois JS, Perkins EM (2015). Aub and Ago3 are recruited to nuage through two mechanisms to form a Ping-Pong complex assembled by Krimper. Mol Cell.

[46] Zamore PD (2010). Somatic piRNA biogenesis. EMBO J.

[47] Sai Lakshmi S, Agrawal S (2008). piRNABank: a web resource on classified and clustered Piwi-interacting RNAs. Nucleic Acids Res.

[48] Kozomara A, Griffiths-Jones S (2014). miRBase: annotating high confidence microRNAs using deep sequencing data. Nucleic Acids Res.

[49] Yang Q, Li R, Lyu Q, Hou L, Liu Z, Sun Q (2019). Single-cell CAS-seq reveals a class of short PIWI-interacting RNAs in human oocytes. Nat Commun.

[50] Ernst C, Odom DT, Kutter C (2017). The emergence of piRNAs against transposon invasion to preserve mammalian genome integrity. Nat Commun.

[51] Zhang S, Pointer B, Kelleher ES (2020). Rapid evolution of piRNA-mediated silencing of an invading transposable element was driven by abundant de novo mutations. Genome Res.

[52] Burns KH (2017). Transposable elements in cancer. Nat Rev Cancer.

[53] Payer LM, Burns KH (2019). Transposable elements in human genetic disease. Nat Rev Genet.

[54] Castel SE, Martienssen RA (2013). RNA interference in the nucleus: roles for small RNAs in transcription, epigenetics and beyond. Nat Rev Genet.

[55] De Fazio S, Bartonicek N, Di Giacomo M, Abreu-Goodger C, Sankar A, Funaya C (2011). The endonuclease activity of Mili fuels piRNA amplification that silences LINE1 elements. Nature.

[56] Sienski G, Donertas D, Brennecke J (2012). Transcriptional silencing of transposons by Piwi and maelstrom and its impact on chromatin state and gene expression. Cell.

[57] Neganova ME, Klochkov SG, Aleksandrova YR, Aliev G (2020). Histone modifications in epigenetic regulation of cancer: perspectives and achieved progress. Semin Cancer Biol.

[58] Ninova M, Chen YA, Godneeva B, Rogers AK, Luo Y, Fejes Toth K (2020). Su(var)2–10 and the SUMO pathway link piRNA-guided target recognition to chromatin silencing. Mol Cell.

[59] Siddiqi S, Matushansky I (2012). Piwis and piwi-interacting RNAs in the epigenetics of cancer. J Cell Biochem.

[60] Zoch A, Auchynnikava T, Berrens RV, Kabayama Y, Schopp T, Heep M (2020). SPOCD1 is an essential executor of piRNA-directed de novo DNA methylation. Nature.

[61] Yan H, Wu QL, Sun CY, Ai LS, Deng J, Zhang L (2015). piRNA-823 contributes to tumorigenesis by regulating de novo DNA methylation and angiogenesis in multiple myeloma. Leukemia.

[62] Ai L, Mu S, Sun C, Fan F, Yan H, Qin Y (2019). Myeloid-derived suppressor cells endow stem-like qualities to multiple myeloma cells by inducing piRNA-823 expression and DNMT3B activation. Mol Cancer.

[63] Su JF, Zhao F, Gao ZW, Hou YJ, Li YY, Duan LJ (2020). piR-823 demonstrates tumor oncogenic activity in esophageal squamous cell carcinoma through DNA methylation induction via DNA methyltransferase 3B. Pathol Res Pract.

[64] Reuter M, Berninger P, Chuma S, Shah H, Hosokawa M, Funaya C (2011). Miwi catalysis is required for piRNA amplification-independent LINE1 transposon silencing. Nature.

[65] Wang W, Han BW, Tipping C, Ge DT, Zhang Z, Weng Z (2015). Slicing and binding by Ago3 or Aub trigger piwi-bound piRNA production by distinct mechanisms. Mol Cell.

[66] Peng L, Song L, Liu C, Lv X, Li X, Jie J (2016). piR-55490 inhibits the growth of lung carcinoma by suppressing mTOR signaling. Tumour Biol.

[67] Liu X, Zheng J, Xue Y, Yu H, Gong W, Wang P (2018). PIWIL3/OIP5-AS1/miR-367-3p/CEBPA feedback loop regulates the biological behavior of glioma cells. Theranostics.

[68] Yin J, Jiang XY, Qi W, Ji CG, Xie XL, Zhang DX (2017). piR-823 contributes to colorectal tumorigenesis by enhancing the transcriptional activity of HSF1. Cancer Sci.

[69] Hanahan D, Weinberg RA (2000). The hallmarks of cancer. Cell.

[70] Hanahan D, Weinberg RA (2011). Hallmarks of cancer: the next generation. Cell.

[71] Cheng J, Deng H, Xiao B, Zhou H, Zhou F, Shen Z (2012). piR-823, a novel non-coding small RNA, demonstrates in vitro and in vivo tumor suppressive activity in human gastric cancer cells. Cancer Lett.

[72] Li B, Hong J, Hong M, Wang Y, Yu T, Zang S (2019). piRNA-823 delivered by multiple myeloma-derived extracellular vesicles promoted tumorigenesis through re-educating endothelial cells in the tumor environment. Oncogene.

[73] Reeves ME, Firek M, Jliedi A, Amaar YG (2017). Identification and characterization of RASSF1C piRNA target genes in lung cancer cells. Oncotarget.

[74] Fu A, Jacobs DI, Hoffman AE, Zheng T, Zhu Y (2015). PIWI-interacting RNA 021285 is involved in breast tumorigenesis possibly by remodeling the cancer epigenome. Carcinogenesis.

[75] Singh G, Roy J, Rout P, Mallick B (2018). Genome-wide profiling of the PIWI-interacting RNA-mRNA regulatory networks in epithelial ovarian cancers. PLoS ONE.

[76] Spilka R, Ernst C, Mehta AK, Haybaeck J (2013). Eukaryotic translation initiation factors in cancer development and progression. Cancer Lett.

[77] Laplante M, Sabatini DM (2012). mTOR signaling in growth control and disease. Cell.

[78] Ediriweera MK, Tennekoon KH, Samarakoon SR (2019). Role of the PI3K/AKT/mTOR signaling pathway in ovarian cancer: biological and therapeutic significance. Semin Cancer Biol.

[79] Ma H, Wang H, Tian F, Zhong Y, Liu Z, Liao A (2020). PIWI-interacting RNA-004800 is regulated by S1P receptor signaling pathway to keep myeloma cell survival. Front Oncol.

[80] Law PT, Qin H, Ching AK, Lai KP, Co NN, He M (2013). Deep sequencing of small RNA transcriptome reveals novel non-coding RNAs in hepatocellular carcinoma. J Hepatol.

[81] Burke PJ (2017). Mitochondria, bioenergetics and apoptosis in cancer. Trends Cancer.

[82] Mohammad RM, Muqbil I, Lowe L, Yedjou C, Hsu HY, Lin LT (2015). Broad targeting of resistance to apoptosis in cancer. Semin Cancer Biol.

[83] Wong RS (2011). Apoptosis in cancer: from pathogenesis to treatment. J Exp Clin Cancer Res.

[84] Bai L, Wang S (2014). Targeting apoptosis pathways for new cancer therapeutics. Annu Rev Med.

[85] Lee YJ, Moon SU, Park MG, Jung WY, Park YK, Song SK (2016). Multiplex bioimaging of piRNA molecular pathway-regulated theragnostic effects in a single breast cancer cell using a piRNA molecular beacon. Biomaterials.

[86] Chu H, Hui G, Yuan L, Shi D, Wang Y, Du M (2015). Identification of novel piRNAs in bladder cancer. Cancer Lett.

[87] Das B, Roy J, Jain N, Mallick B (2019). Tumor suppressive activity of PIWI-interacting RNA in human fibrosarcoma mediated through repression of RRM2. Mol Carcinog.

[88] Das B, Jain N, Mallick B (2020). piR-39980 promotes cell proliferation, migration and invasion, and inhibits apoptosis via repression of SERPINB1 in human osteosarcoma. Biol Cell.

[89] Busch J, Ralla B, Jung M, Wotschofsky Z, Trujillo-Arribas E, Schwabe P (2015). Piwi-interacting RNAs as novel prognostic markers in clear cell renal cell carcinomas. J Exp Clin Cancer Res.

[90] Zhang L, Meng X, Li D, Han X (2020). piR-001773 and piR-017184 promote prostate cancer progression by interacting with PCDH9. Cell Signal.

[91] Nishida N, Yano H, Nishida T, Kamura T, Kojiro M (2006). Angiogenesis in cancer. Vasc Health Risk Manag.

[92] Dong Y, Tu R, Liu H, Qing G (2020). Regulation of cancer cell metabolism: oncogenic MYC in the driver's seat. Signal Transduct Target Ther.

[93] Xu X, Han L, Yang H, Duan L, Zhou B, Zhao Y (2013). The A/G allele of eIF3a rs3740556 predicts platinum-based chemotherapy resistance in lung cancer patients. Lung Cancer.

[94] Roy J, Anand K, Mohapatra S, Nayak R, Chattopadhyay T, Mallick B (2020). Single nucleotide polymorphisms in piRNA-pathway genes: an insight into genetic determinants of human diseases. Mol Genet Genom.

[95] Toth KF, Pezic D, Stuwe E, Webster A (2016). The piRNA pathway guards the germline genome against transposable elements. Adv Exp Med Biol.

[96] Zhang W, Liu H, Yin J, Wu W, Zhu D, Amos CI (2016). Genetic variants in the PIWI-piRNA pathway gene DCP1A predict melanoma disease-specific survival. Int J Cancer.

[97] Chu H, Xia L, Qiu X, Gu D, Zhu L, Jin J (2015). Genetic variants in noncoding PIWI-interacting RNA and colorectal cancer risk. Cancer.

[98] Banimohamad-Shotorbani B, Kahroba H, Sadeghzadeh H, Wilson DM, Maadi H, Samadi N (2020). DNA damage repair response in mesenchymal stromal cells: From cellular senescence and aging to apoptosis and differentiation ability. Ageing Res Rev.

[99] Lenart P, Novak J, Bienertova-Vasku J (2018). PIWI-piRNA pathway: setting the pace of aging by reducing DNA damage. Mech Ageing Dev.

[100] Ladabaum U, Dominitz JA, Kahi C, Schoen RE (2020). Strategies for colorectal cancer screening. Gastroenterology.

[101] Tanoue LT, Tanner NT, Gould MK, Silvestri GA (2015). Lung cancer screening. Am J Respir Crit Care Med.

[102] Patel S, Issaka RB, Chen E, Somsouk M (2021). Colorectal cancer screening and COVID-19. Am J Gastroenterol.

[103] Nassar FJ, Nasr R, Talhouk R (2017). MicroRNAs as biomarkers for early breast cancer diagnosis, prognosis and therapy prediction. Pharmacol Ther.

[104] Mai D, Zheng Y, Guo H, Ding P, Bai R, Li M (2020). Serum piRNA-54265 is a new biomarker for early detection and clinical surveillance of human colorectal cancer. Theranostics.

[105] Gu X, Wang C, Deng H, Qing C, Liu R, Liu S (2020). Exosomal piRNA profiling revealed unique circulating piRNA signatures of cholangiocarcinoma and gallbladder carcinoma. Acta Biochim Biophys Sin (Shanghai).

[106] Chang Z, Ji G, Huang R, Chen H, Gao Y, Wang W (2020). PIWI-interacting RNAs piR-13643 and piR-21238 are promising diagnostic biomarkers of papillary thyroid carcinoma. Aging (Albany NY).

[107] Wang Z, Yang H, Ma D, Mu Y, Tan X, Hao Q (2020). Serum PIWI-interacting RNAs piR-020619 and piR-020450 are promising novel biomarkers for early detection of colorectal cancer. Cancer Epidemiol Biomark Prev.

[108] Zhang H, Ren Y, Xu H, Pang D, Duan C, Liu C (2013). The expression of stem cell protein Piwil2 and piR-932 in breast cancer. Surg Oncol.

[109] Qu A, Wang W, Yang Y, Zhang X, Dong Y, Zheng G (2019). A serum piRNA signature as promising non-invasive diagnostic and prognostic biomarkers for colorectal cancer. Cancer Manag Res.

[110] Zhao C, Tolkach Y, Schmidt D, Toma M, Muders MH, Kristiansen G (2019). Mitochondrial PIWI-interacting RNAs are novel biomarkers for clear cell renal cell carcinoma. World J Urol.

[111] Wang Y, Gable T, Ma MZ, Clark D, Zhao J, Zhang Y (2017). A piRNA-like small RNA induces chemoresistance to cisplatin-based therapy by inhibiting apoptosis in lung squamous cell carcinoma. Mol Ther Nucleic Acids.

[112] Jacobs DI, Qin Q, Fu A, Chen Z, Zhou J, Zhu Y (2018). piRNA-8041 is downregulated in human glioblastoma and suppresses tumor growth in vitro and in vivo. Oncotarget.

[113] Huang G, Hu H, Xue X, Shen S, Gao E, Guo G (2013). Altered expression of piRNAs and their relation with clinicopathologic features of breast cancer. Clin Transl Oncol.

[114] Vychytilova-Faltejskova P, Stitkovcova K, Radova L, Sachlova M, Kosarova Z, Slaba K (2018). Circulating PIWI-interacting RNAs piR-5937 and piR-28876 are promising diagnostic biomarkers of colon cancer. Cancer Epidemiol Biomark Prev.

[115] Iliev R, Fedorko M, Machackova T, Mlcochova H, Svoboda M, Pacik D (2016). Expression levels of PIWI-interacting RNA, piR-823, are deregulated in tumor tissue, blood serum and urine of patients with renal cell carcinoma. Anticancer Res.

[116] Zou AE, Zheng H, Saad MA, Rahimy M, Ku J, Kuo SZ (2016). The non-coding landscape of head and neck squamous cell carcinoma. Oncotarget.

[117] Muller S, Raulefs S, Bruns P, Afonso-Grunz F, Plotner A, Thermann R (2015). Next-generation sequencing reveals novel differentially regulated mRNAs, lncRNAs, miRNAs, sdRNAs and a piRNA in pancreatic cancer. Mol Cancer.

[118] Shen S, Yu H, Liu X, Liu Y, Zheng J, Wang P (2018). PIWIL1/piRNA-DQ593109 regulates the permeability of the blood-tumor barrier via the MEG3/miR-330-5p/RUNX3 Axis. Mol Ther Nucleic Acids.

[119] Rounge TB, Furu K, Skotheim RI, Haugen TB, Grotmol T, Enerly E (2015). Profiling of the small RNA populations in human testicular germ cell tumors shows global loss of piRNAs. Mol Cancer.

[120] Gotwals P, Cameron S, Cipolletta D, Cremasco V, Crystal A, Hewes B (2017). Prospects for combining targeted and conventional cancer therapy with immunotherapy. Nat Rev Cancer.

[121] Wu Q, Yang Z, Nie Y, Shi Y, Fan D (2014). Multi-drug resistance in cancer chemotherapeutics: mechanisms and lab approaches. Cancer Lett.

[122] Galletti G, Leach BI, Lam L, Tagawa ST (2017). Mechanisms of resistance to systemic therapy in metastatic castration-resistant prostate cancer. Cancer Treat Rev.

[123] Darragh LB, Oweida AJ, Karam SD (2018). Overcoming resistance to combination radiation-immunotherapy: a focus on contributing pathways within the tumor microenvironment. Front Immunol.

[124] Ramapriyan R, Caetano MS, Barsoumian HB, Mafra ACP, Zambalde EP, Menon H (2019). Altered cancer metabolism in mechanisms of immunotherapy resistance. Pharmacol Ther.

[125] Zhang X, Xie K, Zhou H, Wu Y, Li C, Liu Y (2020). Role of non-coding RNAs and RNA modifiers in cancer therapy resistance. Mol Cancer.

[126] Cui C, Yang J, Li X, Liu D, Fu L, Wang X (2020). Functions and mechanisms of circular RNAs in cancer radiotherapy and chemotherapy resistance. Mol Cancer.

[127] Iqbal MA, Arora S, Prakasam G, Calin GA, Syed MA (2019). MicroRNA in lung cancer: role, mechanisms, pathways and therapeutic relevance. Mol Aspects Med.

[128] Peng L, Jiang J, Tang B, Nice EC, Zhang YY, Xie N (2020). Managing therapeutic resistance in breast cancer: from the lncRNAs perspective. Theranostics.

[129] Wang K, Liang C, Liu J, Xiao H, Huang S, Xu J (2014). Prediction of piRNAs using transposon interaction and a support vector machine. BMC Bioinform.

[130] Li D, Luo L, Zhang W, Liu F, Luo F (2016). A genetic algorithm-based weighted ensemble method for predicting transposon-derived piRNAs. BMC Bioinform.

[131] Brayet J, Zehraoui F, Jeanson-Leh L, Israeli D, Tahi F (2014). Towards a piRNA prediction using multiple kernel fusion and support vector machine. Bioinformatics.

[132] Liu B, Yang F, Chou KC (2017). 2L-piRNA: a two-layer ensemble classifier for identifying piwi-interacting RNAs and their function. Mol Ther Nucleic Acids.

[133] Liu X, Ding J, Gong F (2014). piRNA identification based on motif discovery. Mol Biosyst.

[134] Boucheham A, Sommard V, Zehraoui F, Boualem A, Batouche M, Bendahmane A (2017). IpiRId: integrative approach for piRNA prediction using genomic and epigenomic data. PLoS ONE.

[135] Pian C, Chen YY, Zhang J, Chen Z, Zhang GL, Li Q (2017). V-ELMpiRNAPred: Identification of human piRNAs by the voting-based extreme learning machine (V-ELM) with a new hybrid feature. J Bioinform Comput Biol.

[136] Rosenkranz D, Zischler H (2012). proTRAC–a software for probabilistic piRNA cluster detection, visualization and analysis. BMC Bioinformatics.

[137] Jung I, Park JC, Kim S (2014). piClust: a density based piRNA clustering algorithm. Comput Biol Chem.

[138] Wang K, Hoeksema J, Liang C (2018). piRNN: deep learning algorithm for piRNA prediction. PeerJ.

[139] Zhang P, Si X, Skogerbo G, Wang J, Cui D, Li Y (2014). piRBase: a web resource assisting piRNA functional study. Database (Oxford).

[140] Wang J, Zhang P, Lu Y, Li Y, Zheng Y, Kan Y (2019). piRBase: a comprehensive database of piRNA sequences. Nucleic Acids Res.

[141] Yuan J, Zhang P, Cui Y, Wang J, Skogerbo G, Huang DW (2016). Computational identification of piRNA targets on mouse mRNAs. Bioinformatics.

[142] Pang KC, Stephen S, Dinger ME, Engstrom PG, Lenhard B, Mattick JS (2007). RNAdb 2.0—an expanded database of mammalian non-coding RNAs. Nucleic Acids Res.

[143] Tosar JP, Rovira C, Cayota A (2018). Non-coding RNA fragments account for the majority of annotated piRNAs expressed in somatic non-gonadal tissues. Commun Biol.

[144] Rosenkranz D (2016). piRNA cluster database: a web resource for piRNA producing loci. Nucleic Acids Res.

[145] Sarkar A, Maji RK, Saha S, Ghosh Z (2014). piRNAQuest: searching the piRNAome for silencers. BMC Genom.

[146] Zhang H, Ali A, Gao J, Ban R, Jiang X, Zhang Y (2018). IsopiRBank: a research resource for tracking piRNA isoforms. Database (Oxford).

[147] Wu WS, Huang WC, Brown JS, Zhang D, Song X, Chen H (2018). pirScan: a webserver to predict piRNA targeting sites and to avoid transgene silencing in C. elegans. Nucleic Acids Res.

[148] Wu WS, Brown JS, Chen TT, Chu YH, Huang WC, Tu S (2019). piRTarBase: a database of piRNA targeting sites and their roles in gene regulation. Nucleic Acids Res.

[149] Xin J, Du M, Jiang X, Wu Y, Ben S, Zheng R (2021). Systematic evaluation of the effects of genetic variants on PIWI-interacting RNA expression across 33 cancer types. Nucleic Acids Res.

[150] Jansson MD, Lund AH (2012). MicroRNA and cancer. Mol Oncol.

[151] Meng S, Zhou H, Feng Z, Xu Z, Tang Y, Li P (2017). CircRNA: functions and properties of a novel potential biomarker for cancer. Mol Cancer.

[152] Gou LT, Dai P, Yang JH, Xue Y, Hu YP, Zhou Y (2014). Pachytene piRNAs instruct massive mRNA elimination during late spermiogenesis. Cell Res.

[153] Dai P, Wang X, Gou LT, Li ZT, Wen Z, Chen ZG (2019). A Translation-activating function of MIWI/piRNA during mouse spermiogenesis. Cell.

[154] Shi S, Yang ZZ, Liu S, Yang F, Lin H (2020). PIWIL1 promotes gastric cancer via a piRNA-independent mechanism. Proc Natl Acad Sci U S A.

[155] Li F, Yuan P, Rao M, Jin CH, Tang W, Rong YF (2020). piRNA-independent function of PIWIL1 as a co-activator for anaphase promoting complex/cyclosome to drive pancreatic cancer metastasis. Nat Cell Biol.

